# Usefulness of Echocardiographic Parameters of Myocardial Work in Patients with Aortic Stenosis Undergoing Transcatheter Aortic Valve Implantation

**DOI:** 10.3390/jcm14020512

**Published:** 2025-01-15

**Authors:** Anna Polewczyk, Edward Pietrzyk, Maciej Polewczyk, Dariusz Jarek, Dariusz Dudek

**Affiliations:** 1 Institute of Medical Sciences, Jan Kochanowski University, 25-369 Kielce, Poland; edward.pietrzyk_xl@wp.pl (E.P.); maciek.polewczyk@gmail.com (M.P.); 2 Cardiac Surgery Department, Provincial Hospital in Kielce, 25-736 Kielce, Poland; dyarok@wp.pl; 3 Acute Cardiac Care Unit, Provincial Hospital in Kielce, 25-736 Kielce, Poland; 4 2nd Department of Cardiology, Jagiellonian University Medical College, 31-008 Krakow, Poland; mcdudek@cyf-kr.edu.pl

**Keywords:** myocardial work, aortic stenosis, transcatheter aortic valve implantation

## Abstract

**Background:** Myocardial work (MW) is a new echocardiographic parameter used in the assessment of cardiac energy expenditure. The aim of the current study was to evaluate changes in left ventricular MW parameters in patients with severe aortic stenosis undergoing transcatheter aortic valve implantation (TAVI). **Methods:** One hundred and thirty five consecutive patients who underwent TAVI at one center were evaluated before and after the procedure using transthoracic echocardiography (TTE) to assess the following MW indices: global constructive work (GCW), global wasted work (GWW), global work index (GWI) and global work efficiency (GWE). **Results:** The comparison of MW parameters before and an average of 5.9 days after TAVI showed an increase in GCW, GWW and GWI, and no significant change in GWE. A detailed analysis showed an increase in GCW and GWI only in patients with the worst initial global longitudinal strain (GLS) > −8.0%: 845.2 vs. 852.2; *p* < 0.001 and 469.7 vs. 499.0 mmHg%; *p* < 0.001, respectively, whereas in the group of patients with GLS < −16.0%, a reduction in these indices was observed: 2135.8 vs. 2043.0; *p* < 0.001 and 1732.4 vs. 1633.1 mmHg%; *p* < 0.001. The significant increase in GWE was observed in patients with left ventricular ejection fraction (LVEF) < 30%: 77.7 vs. 72.0; *p* = 0.043 and GLS > −8.0%: 74.4 vs. 71.0 mmHg%; *p* < 0.001. The increase in GCW and GWI parameters after TAVI was strongly correlated with LVEF and pressure aortic gradient (PGA) before the procedure. **Conclusions:** Echocardiographic assessment of myocardial work parameters is a valuable method of documenting hemodynamic changes in patients with severe aortic stenosis before and after TAVI. Long-term left ventricular overload in patients with aortic stenosis results in a global reduction of myocardial work parameters; therefore, in patients with the lowest LVEF and GLS, the increased GCW, GWI and GWE reflect energy reserves enabling a rapid increase in the effective work of the heart.

## 1. Introduction

Myocardial work (MW) is a relatively new method for assessing left ventricular (LV) function. MW is measured from pressure-strain loops (PSL), which are constructed from LV pressure curves combined with global longitudinal strain (GLS) [1]. Previous studies seem to confirm that in many clinical situations, MW is a more sensitive parameter in the assessment of left ventricular myocardial dysfunction than left ventricular ejection fraction (LVEF) [2,3,4,5,6]. Early detection of left ventricular dysfunction in patients with valvular defects is of great importance in accelerating qualification for surgical treatment. The most common valvular disease in the developed world is aortic stenosis (AS) [7,8,9]. Current guidelines recommend surgical or transcatheter aortic valve replacement/implantation (SAVR or TAVI) in severe AS with symptoms, or in asymptomatic patients with LVEF <50% [7]. Since LVEF is not a sufficiently sensitive marker, there are other parameters used in multimodality imaging techniques, including longitudinal strain, exercise stress echo and cardiac magnetic resonance imaging (MRI). These examinations offer potentially better ways to evaluate patients, schedule surgery and predict recovery [7]. The aim of the study was to assess the possibility of using new echocardiographic parameters—MW in the early detection of left ventricular dysfunction in patients with severe AS and to assess the benefits after TAVI.

## 2. Methods

### 2.1. Study Population

The study was based on data from 135 consecutive patients who underwent TAVI for severe aortic stenosis in a single center between April 2021 and August 2023. All patients were qualified for the procedure according to ESC guidelines [7], following the Heart Team Decision.

### 2.2. Echocardiographic Analysis

All patients underwent transthoracic echocardiographic (TTE) examinations on average 1.5 days before and 5.9 days after the procedure. TTE was performed using a E95-GE machine (GE Healthcare, Horten, Norway) with complete assessment of standard parameters: chamber dimensions, systolic left ventricular function (LVEF) utilizing Simpson’s biplane methods, diastolic LV function, left atrial volume index (LAVI), LV outflow tract (LVOT) stroke volume and valvular function with the assessment of aortic valve (AV) peak velocity, AV maximum and mean paravalvular gradient (PGA), and AV area (AVA) derived from the simplified Bernoulli equation, according to current recommendations [10,11]. All examinations were analyzed in post-processing using semi-automatic software (EchoPAC, ver. 204, Horten, Norway) to obtain LV global longitudinal strain (GLS) and parameters of myocardial work.

Global longitudinal strain was determined from apical images by maximizing the frame rate (from 50 to 70 frames per second) and by narrowing the sector to isolate individual walls. Apical 4-chamber, 3-chamber and 2-chamber views were obtained to assess the longitudinal strain in each wall at the basal, mid and apical level in each of these views. For each view, three cardiac cycles were recorded and saved digitally for further study. Automatic functional imaging (AFI) automatically tracked the LV endocardial and epicardial boundaries in the three apical dynamic images. Tracking was accepted or rejected according to the quality of image. The global longitudinal strain of the left ventricle was calculated systematically from the average value of the three views, including 18 segments of the myocardium.

The parameters of myocardial work include global constructive work (GCW), global wasted work (GWW), global work index (GWI) and global effective work (GWE). LV myocardial work was calculated as a function of time throughout the cardiac cycle by the combination of LV strain data obtained by speckle-tracking echocardiography and a non-invasively estimated LV pressure curve. After manually inserting blood pressure measurements, the software automatically generates a noninvasive LV pressure-systolic loop (PSL) created from the duration of the isovolumic and ejection phases defined by valve timing events that were determined by the opening and closing of the mitral and aortic valves. GWI was defined as myocardial work within the area of the LV PSL calculated from mitral valve closure to mitral valve opening, and GCW as work contributing to LV ejection. GWW was defined as work that does not contribute to LV ejection. GWE was calculated as the ratio of constructive work to total work (both constructive and wasted).

### 2.3. Data Analysis

The study evaluated myocardial work indices and their relationship with two-dimensional echocardiographic parameters: left ventricular ejection fraction, two-dimensional speckle tracking (2DSTE), left ventricular global longitudinal strain (LVGLS), and peak and mean aortic valve gradient measured by Doppler velocities before and after transcatheter aortic valve implantation. In addition, LVGLS was stratified into three and LVEF into four categories of systolic contractility to further examine LV myocardial work. All analyses were performed offline using semi-automated software (Version 204). Correlation analysis was performed to assess the relationship between MW, GCW, GWW, GWI, and GWE indices, and LVEF and LVGLS.

### 2.4. Consent

Written informed consent was obtained from all patients involved in the study.

The study protocol and consent to publication were approved by the Bioethics Committee at the Regional Chamber of Physicians no. 59/2021.

### 2.5. Statistical Analysis

To compare the selected clinical parameters and mean LVEF, mean GLS and average max and mean PGA in the entire study population before and after TAVI, the Student’s *t*-test for two matched samples was used, and in the case of lack of normality in the analyzed variable distributions, the Wilcoxon paired order test was used. Spearman’s or Pearson’s correlation analysis was performed to examine the relationship between LVEF, GLS, GCW, GWW, GWI and GWE before and after TAVI with selected echocardiographic parameters. Spearman’s or Pearson’s correlation was also used to analyze changes in the GWE parameter before and after the TAVI procedure in patients with specific LVEF ranks.

The Kruskal–Wallis test was used to analyze changes in MW parameters before and after the TAVI procedure in patients with specific GLS ranks.

A significance level of α = 0.05 was assumed in all statistical tests. Statistical analysis was done using the STATISTICA ver 13.1 advanced analytics software.

## 3. Results

From April 2021 to August 2023, a total of 135 patients with severe AS (mean age 78.9 years; 48.9% women) were enrolled. The mean LVEF of patients undergoing TAVI was 49.5% ± 12.8%, and mean GLS was −10.5 ± 3.8. More than 70% of patients presented with heart failure in NYHA class III–IV, 81.5% had hypertension, and 63.7% were treated for ischemic heart disease, with history of percutaneous coronary angioplasty (PCI) in 17.8% and coronary artery bypass graft (CABG) in 8.9% of patients. Permanent AF occurred in 25.2% of patients. Diabetes was diagnosed in 36.3% of patients, chronic kidney disease in 17.0% of patients, and chronic obstructive pulmonary disease (COPD) in 12.6% of patients (Table 1).

LVEF > 50% was observed in 60% of the study patients. Most patients (39.3%) had GLS of ranging from −12.0 to −8.0. GLS < −16.0 was present in 8.2% of patients. GLS > −8.0 was observed in 27.4% of patients. In 93.3% of patients AVA was <0.5 cm^2^. Maximum PGA above 80 mmHg was observed in 71 (52.6%) of patients, and mean PGA above 40 mmHg was found in 111 (82.2%) of patients. Severe aortic regurgitation (AR III–IV grade) was observed in 17 (12.6%) of patients (Table 2).

**Figure 1:**
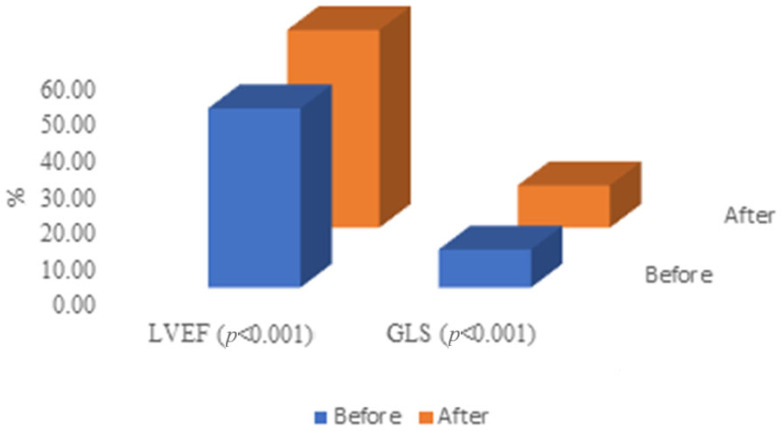
Comparison of LVEF and GLS before and after TAVI. GLS—global longitudinal strain, LVEF—left ventricular ejection fraction.

**Figure 2:**
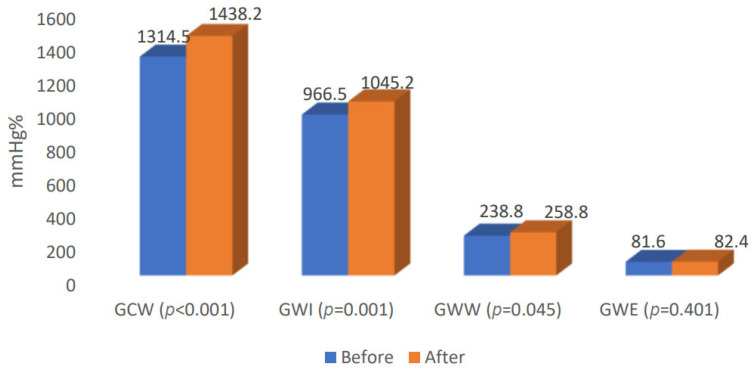
Comparison of MW parameters before and after TAVI. GCW—global constructive work, GWI—global work index, GWW—global waste work, GWE—global work efficiency.

A comparison of echocardiographic examinations before and after TAVI showed a significant increase in some parameters: the mean LVEF increased by 5.2, mean GLS increased by 1.2, mean GCW increased by 141.5, mean GWI increased by 102.0 and mean GWW increased by 20.0. The GWE parameter was comparable before and after TAVI (Table 3, Figure 1 and Figure 2).

An analysis of parameters affecting GWE showed that a significant increase in the GWE was observed only in patients with LVEF below 30% (Table 4).

An analysis of changes in MW parameters in individual GLS intervals (R1—GLS < −16.0%, R2—GLS from −16.0% to −8.0% and R3—GLS > −8.0%) showed a decrease in GCW after TAVI in patients with GLS < −16.0% and an increase in GCW after TAVI in groups R2 and R3.

A comparison of changes in GWI parameters in individual GLS intervals (R1, R2, R3) showed a decrease in GWI in patients in intervals R1 and R2 and an increase in GWI in interval R3.

An analysis of GWW before TAVI showed the lowest values of this parameter in patients with GLS < −16.0%; the highest GWW values were observed in the GLS > −8.0% group. Changes in GWW before and after TAVI for individual GLS intervals were statistically insignificant.

The GWE parameter before TAVI was the highest in patients with GLS < −16.0% and the lowest in the GLS > −8.0% group. Analysis of GWE changes before and after TAVI showed a significant reduction of this parameter in patients from groups R1 and R2 and a significant increase in GWE in group R3 (Table 5).

The analysis of the relationship between individual MW components and other echocardiographic parameters in patients with AS before TAVI showed the positive correlation of GCW and LVEF and max PGA. The assessment of the relationship between GWW and other echocardiographic parameters did not reveal any significant correlations. The analysis of the relationship of GWI and GWE with other echocardiographic parameters showed positive correlation with LVEF and max and mean PGA, and negative correlation with mitral regurgitation (Table 6).

The analysis of the relationship between individual MW components after TAVI and other echocardiographic parameters before TAVI showed the correlation between GCW and GWI and LVEF and max and mean PGA. There was no correlation between GWW after TAVI and the analyzed echocardiographic parameters before TAVI. GWE after TAVI correlated with LVEF before TAVI (Table 7).

## 4. Discussion

The main findings showed a good positive correlation before TAVI between GCW, GWI and GWE and LVEF, which remains after TAVI with the exception of GWE. In the entire study population, GLS and LVEF increased after TAVI. MW indices also increased after TAVI, except for GWE, which showed no difference before and after the procedure. The results showed a statistically significant increase in GWE only in patients with LVEF ≤30% and GLS > −8%, while GWI increased significantly in patients with GLS < −8%. GCW decreased only in the group of patients with GLS < −16%, GWW increased in the group with GLS <−16% and in the group with GLS from −16 to −12%, while it decreased in the group >−8%.

Echocardiography is a key diagnostic method for the degree of aortic stenosis, enabling the assessment of valve morphology and left ventricular function and providing prognostic information. Assessment of left ventricular systolic function using LVEF is considered the most important parameter for the timing of intervention. However, LVEF is often preserved until late in the disease course, even after the onset of clinical symptoms resulting from a progression of AS severity and left ventricular hypertrophy, indicating the lack of accuracy of this parameter in detecting discrete changes in myocardial function [12]. In recent years, the new echocardiographic parameters GLS and MW have been increasingly used in the assessment of left ventricular function. GLS in patients with severe aortic stenosis appears to be a more sensitive parameter of subclinical left ventricular dysfunction. In the current study, 60% of patients had normal LVEF ≥50%, while decreased GLS was found in 89% of subjects. These results are consistent with other reports: in a population of 411 patients with symptomatic severe AS treated with TAVI, LVEF was preserved in 60% of patients, while impaired GLS was seen in 75% of the patients [13]. Thus, GLS enables a more precise evaluation of left ventricular function in patients with aortic stenosis; however, as is known, both LVEF and GLS are parameters dependent on afterload and they do not provide information regarding the efficiency of the ventricle. Workload is a key factor influencing quantitative parameters of LV systolic function. MW enables the assessment of left ventricular function in a way that incorporates afterload and allows for the quantification of energy losses [1,13,14]. The classic pressure–volume loop, derived from invasive hemodynamics, forms the basis of our understanding of the contributions of preload, afterload, and contractility to left ventricular systolic function. The “area” in this loop is referred to as left ventricular stroke work and was the first way to conceptualize MW. Subsequently, the pressure–strain loop and MW indices have emerged, which offer a complementary picture of left ventricular systolic function [15]. Myocardial work may help us clarify two severe AS echocardiographic phenotypes: (1) patients who have abnormal GLS but high myocardial work (in whom the myocardium is able to compensate for increased afterload with increased work) and (2) those with abnormal GLS and reduced (relative to afterload) myocardial work (who may have already experienced sustained adverse LV remodeling) [16]. In the present study, patients with severe aortic stenosis who qualified for TAVI had initially very low GCW, GWI, GWE and high GWW in comparison to healthy subjects assessed in a contemporary metanalysis [17] (GCW: 1314 mmHg% vs. 2278 mmHg%, GWI: 967 mmHg% vs. 2010 mmHg%, GWE: 82% vs. 96%) GWW: 239 mmHg% vs. 80 mmHg%). The current study also demonstrated that the individual MW parameters GCW, GWI and GWE in patients with severe aortic stenosis correlated with LVEF and the value of the transvalvular gradient. As is known, adaptive remodeling of the left ventricle is observed in patients with severe aortic stenosis. In the first stage, a longstanding increase in global afterload results in concentric LV hypertrophy. Adaptive remodeling becomes maladaptive with increasing LV hypertrophy and consequent myocardial fibrosis. It is probable that the baseline parameters in patients with AS reflect subclinical heart failure, not confirmed by commonly used echocardiographic parameters [18,19]. The changes in GLS and MW after TAVI presumably reflect the degree of reversibility of left ventricular damage. The present study showed a significant increase in GLS, GCW, GWI and GWW quickly after the procedure. The data are inconsistent with those presented in few of the previous publications. A study based on data from 35 patients showed an increase in GLS and a reduction in GCW and GWI immediately after TAVI; however, such results were observed for corrected MW parameters (using estimated invasively LV systolic pressure) [20]. Another study evaluating a group of 125 patients also showed a reduction in GCW and GWI parameters, as well as a reduction in GWE, and no improvement in LVEF and GLS [21]. Similarly, based on the analysis of MW before and after TAVI in a group of 53 patients, a decrease in all MW parameters was found after the procedure [22]. Such significant differences in the results of the current study and previous data from the literature are probably due to the heterogeneous study population. In the present study, GLS and MW parameters before TAVI were very low in comparison to other analysis [20,21,22]. Moreover, a detailed analysis of changes in MW parameters after TAVI showed that a significant increase in GCW and GWI was observed only in patients with significantly reduced GLS (>−8%) at baseline. The very low MW parameters in patients with severe AS probably result from long-lasting ischemia, causing stunning of the myocardium, and the rapid improvement after TAVI results in immediate activation of contractile reserves. It is also very important to analyze the relationship between individual MW parameters. Previous studies have not shown any obvious improvement in the efficiency of the left ventricle—GWE did not change significantly after TAVI [20,21,22]. The present study demonstrated for the first time a significant increase in GWE in patients with very low LVEF (≤30%) and very low GLS value (>−8%). This seems to confirm that MW parameters reflect well the hemodynamic changes occurring in patients with aortic stenosis undergoing TAVI and allow for the assessment of the reversibility of left ventricular damage. This phenomenon is probably more significant in patients who have experienced severe myocardial stunning/freezing due to chronic ischemia associated with severe AS.

The current study also demonstrated that the individual MW parameters GCW, GWI and GWE in patients with severe aortic stenosis correlated with LVEF and the value of the transvalvular gradient. A similar correlation was demonstrated for these parameters after TAVI. Additionally, GCW before TAVI negatively correlated with mitral regurgitation. This means that in patients with very high left ventricular overload due to severe aortic stenosis, the compensatory mechanisms are exhausted and the efficiency of the left ventricle decreases, while after its sudden unloading, we observe a significant increase in myocardial work parameters. The role of the GWW parameter requires a separate explanation in the assessment of hemodynamic changes in patients with severe AS. In the current study, high GWW values were observed before TAVI, and they increased further after the procedure. This probably reflects left ventricular muscle remodeling with a large amount of energy wasted on increasing LV contraction, which becomes increasingly ineffective over time. Perhaps maintaining this parameter after TAVI requires longer follow-up.

## 5. Limitations

This is a single-center study based on a relatively small number of patients. There were no invasive hemodynamic measurements in the study.

## 6. Conclusions

Echocardiographic indices of myocardial work may be useful in assessing hemodynamic changes in patients with severe aortic valve stenosis and in evaluating the improvement of left ventricular function after TAVI. The frequently observed significant reduction in GLS and MW components in patients with severe AS with preserved left ventricular ejection fraction proves that these parameters are more sensitive than LVEF in detecting left ventricular dysfunction in this group of patients. The increase in MW indices in patients with the worst initial parameters reflects the ability to quickly mobilize energy reserves after a sudden increase in flow through the aortic valve. Improving the efficiency of the left ventricle is particularly important in patients with initially very severe systolic dysfunction.

## Figures and Tables

**Table 1:** Clinical characteristics of study group.

Parameters	Value
Number of patients	135
Age (years) (mean, SD)	78.9 ± 7.2
Females (n, %)	66 (48.9%)
BMI mean (kg/m^2^)	27.0
LVEF (%) (mean, SD)	49.5 ± 12.8
GLS (%) (mean, SD)	−10.5 ± 3.8
Max PGA (mean, SD)	Mean 84.4, SD 26.1
Mean PGA (mean, SD)	Mean 55.3, SD 18.1
Hypertension (n, %)	110 (81.5%)
Ischaemic heart diseases (n, %)	86 (63.7%)
History of PCI (n, %)	24 (17.8%)
History of CABG (n, %)	12 (8.9%)
Diabetes (n, %)	49 (36.3%)
AF permament (n, %)	34 (25.2%)
Chronic kidney disease (creatinine level > 1.5 mg/dL) (n, %)	23 (17.0%)
COPD	17 (12.6%)

AF—atrial fibrillation, BMI—body mass index, CABG—coronary artery bypass graft, COPD—chronic obstructive pulmonary disease, GLS—global longitudinal strain, LVEF—left ventricular ejection fraction, PGA—pressure gradient across aortic valve.

**Table 2:** Detailed echocardiographic parameters assessed before TAVI.

Parameters	Number of Patients
LVEF ≥ 50% (n, %)	81 (60.0%)
LVEF 41–49% (n, %)	17 (12.6%)
LVEF ≤ 40% (n, %)	21 (15.6%)
LVEF ≤ 30% (n, %)	16 (11.9%)
GLS < −16.0 (n, %)	11 (8.2%)
GLS from−16.0 to −12.0 (n, %)	34 (25.2%)
GLS from −11.9 to −8.0 (n, %)	53 (39.3%)
GLS > −8.0 (n, %)	37 (27.4%)
AVA < 0.5 cm^2^/m^2^ (n, %)	126 (93.3%)
Max PGA > 80 mmHg (mean, SD)	71 (52.6%)
Mean PGA > 40 mmHg (mean, SD)	111 (82.2%)
AR III grade (n, %)	15 (11.1%)
AR IV grade (n, %)	2 (1.5%)

AR—aortic regurgitation, AVA—aortic valve area, GLS—global longitudinal strain, LVEF—left ventricular ejection fraction, PGA—pressure gradient across aortic valve.

**Table 3:** Echocardiographic parameters analyzed before and after TAVI.

Parameters	Before TAVI	After TAVI	*p*-Value
LVEF (%)	49.5 ± 12.8	54.6 ± 11.2	<0.001 ^A^
GLS (%)	−10.5 ± 3.8	−11.7 ± 3.6	<0.001 ^A^
GCW (mmHg%)	1314.5 ± 487.5	1438.2 ± 468.9	<0.001 ^A^
GWI (mmHg%)	966.5 ± 456.1	1045.2 ± 428.7	0.001 ^A^
GWW (mmHg%)	238.8 ± 160.7	258.8 ± 145.1	0.045 ^B^
GWE (%)	81.6 ± 10.5	82.5 ± 7.8	0.401 ^B^

^A^ Student’s *t*-test for dependent samples. ^B^ Wilcoxon signed-ranked test, *p* < α; α = 0.05. LVEF—left ventricular ejection fraction, GLS—global longitudinal strain, GCW—global constructive work, GWI—global work index, GWW—global waste work, GWE—global work efficiency.

**Table 4:** Changes in the GWE parameter before and after TAVI in patients with particular LVEF ranks.

Variable	GWE Value Mean (SD) Before TAVI	Min–Max Before TAVI	GWE Value Mean (SD) After TAVI	Min–Max After TAVI	*p*-Value
LVEF rank 1	72.0	55–85	77.7	66–90	0.043 ^A^
LVEF rank 2	76.5	42–90	82.6	69–92	0.062 ^A^
LVEF rank 3	81.0	62–93	80.0	54–93	0.662 ^A^
LVEF rank 4	84.9	41–96	83.9	60–95	0.299 ^B^

^A^ Spearman rank correlations, ^B^ Pearson correlation. LVEF—left ventricular ejection fraction, rank 1—LVEF ≤ 30%, rank 2—LVEF 31–40%, rank 3—LVEF 41–49%, rank 4—LVEF ≥ 50%. GWE—global work efficiency, TAVI—transcatheter aortic valve implantation.

**Table 5:** Changes in the MW parameters before and after TAVI in patients with particular GLS ranks.

Variable	MW Parameters Mean (SD)	*p*-Value	Variable	MW Parameters Mean (SD)	*p*-Value
Before TAVI			After TAVI		
GCW					
GLS R1	2135.8 (231.6)	*p* < 0.001 * R1 vs. R2 *p* < 0.001 R1 vs. R3 *p* < 0.001 R2 vs. R3 *p* < 0.001	GLS R1	204.0 (468.9)	*p* < 0.001 * R1 vs. R2 *p* < 0.001 R1 vs. R3 *p* < 0.001 R2 vs. R3 *p* < 0.001
GLS R2	1418.4 (276.2)		GLS R2	1479.1 (361.6)	
GLS R3	845.2 (298.6)		GLS R3	852.2 (239.5)	
GWI					
GLS R1	1732.4 (293.2)	*p* < 0.001 * R1 vs. R2 *p* < 0.001 R1 vs. R3 *p* < 0.001 R2 vs. R3 *p* < 0.001	GLS R1	1633.1 (406.5)	*p* < 0.001 * R1 vs. R2 *p* < 0.001 R1 vs. R3 *p* < 0.001 R2 vs. R3 *p* < 0.001
GLS R2	1088.3 (312.7)		GLS R2	1079.6 (320.0)	
GLS R3	469.7 (252.1)		GLS R3	499.0 (178.5)	
GWW					
GLS R1	178.7 (100.2)	*p* = 0.004 * R2 vs. R3 *p* = 0.044	GLS R1	205.5 (10.7)	*p* = 0.367
GLS R2	210.1 (138.9)		GLS R2	265.0 (149.7)	
GLS R3	323.3 (248.9)		GLS R3	266.8 (144.6)	
GWE					
GLS R1	90.8 (3.2)	*p* < 0.001 * R1 vs. R2 *p* = 0.031 R1 vs. R3 *p* < 0.001 R2 vs. R3 *p* < 0.001	GLS R1	89.5 (3.2)	*p* < 0.001 * R1 vs. R2 *p* = 0.002 R1 vs. R3 *p* < 0.001 R2 vs. R3 *p* < 0.001
GLS R2	85.0 (6.7)		GLS R2	83.2 (6.7)	
GLS R3	71.9 (11.4)		GLS R3	74.4 (8.0)	

* Kruskal–Wallis test. GLS—global longitudinal strain, rank 1—GLS < −16.0%, (R1) rank 2—GLS from −16.0% to −12.0% (R2), rank 3—GLS > −8.0% (R3), GCW—global constructive work, GWI—global work index, GWW—global wasted work, GWE—global work efficiency, TAVI—transcatheter aortic valve implantation.

**Table 6:** Correlation between MW components and other echocardiographic parameters before TAVI.

Parameters MW Before TAVI	Clinical and Echocardiographic Parameters Before TAVI	Correlation Coefficient r	*p*-Value
GCW (mmHg%)	LVEF (%)	0.6	<0.000 ^A^
GCW (mmHg%)	max PGA (mmHg)	0.2	0.039 ^A^
GCW (mmHg%)	mean PGA (mmHg)	0.1	0.274 ^B^
GCW (mmHg%)	degree of AR	0.0	0.600 ^A^
GCW	degree of MR	−0.3	0.003 ^A^
GWW (mmHg%)	LVEF (%)	−0.1	0.097 ^A^
GWW (mmHg%)	max PGA (mmHg)	−0.1	0.103 ^A^
GWW (mmHg%)	mean PGA (mmHg)	−0.1	0.086 ^A^
GWW (mmHg%)	degree of AR	0.0	0.793 ^A^
GWW	degree of MR	−0.1	0.558 ^A^
GWI (mmHg%)	LVEF (%)	0.7	<0.001 ^A^
GWI (mmHg%)	max PGA (mmHg)	0.2	0.010 ^A^
GWI (mmHg%)	mean PGA (mmHg)	0.2	0.011 ^A^
GWI (mmHg%)	degree of AR	0.0	0.858 ^A^
GWI	degree of MR	−0.2	0.005 ^A^
GWE (%)	LVEF (%)	0.5	<0.001 ^A^
GWE (%)	max PGA (mmHg)	0.2	0.005 ^A^
GWE (%)	mean PGA (mmHg)	0.2	0.005 ^A^
GWE (%)	degree of AR	0.0	0.644 ^A^
GWE	degree of MR	−0.1	0.121 ^A^

^A^ Spearman rank correlations, ^B^ Pearson correlation. AR—aortic regurgitation, GCW—global constructive work, GWE—global work efficiency, GWI—global work index, GWW—global waste work, LVEF—left ventricular ejection fraction, PGA—pressure gradient across aortic valve.

**Table 7:** Correlation between MW components after TAVI and other echocardiographic parameters before TAVI.

Parameters MW After TAVI	Clinical and Echocardiographic Parameters Before TAVI	Correlation Coefficient r	*p*-Value
GCW (mmHg%)	LVEF (%)	0.5	<0.001 ^A^
GCW (mmHg%)	max PGA (mmHg)	0.4	<0.001 ^A^
GCW (mmHg%)	mean PGA (mmHg)	0.3	<0.001 ^B^
GCW (mmHg%)	degree of AR	0.0	0.889 ^A^
GCW	degree of MR	−0.1	0.129 ^A^
GWW (mmHg%)	LVEF (%)	0.1	0.196 ^A^
GWW (mmHg%)	max PGA (mmHg)	0.2	0.073 ^A^
GWW (mmHg%)	mean PGA (mmHg)	0.1	0.115 ^A^
GWW (mmHg%)	degree of AR	0.0	0.741 ^A^
GWW	degree of MR	−0.1	0.221 ^A^
GWI (mmHg%)	LVEF (%)	0.5	<0.001 ^A^
GWI (mmHg%)	max PGA (mmHg)	0.4	<0.001 ^A^
GWI (mmHg%)	mean PGA(mmHg)	0.3	0.001 ^B^
GWI (mmHg%)	degree of AR	−0.1	0.474 ^A^
GWI	degree of MR	−0.1	0.137 ^A^
GWE (%)	LVEF (%)	0.3	0.001 ^A^
GWE (%)	max PGA (mmHg)	0.1	0.233 ^A^
GWE (%)	mean PGA (mmHg)	0.1	0.129 ^A^
GWE (%)	degree of AR	−0.1	0.295 ^A^
GWE	degree of MR	0.0	0.643 ^A^

^A^ Spearman rank correlations, ^B^ Pearson correlation. AR—aortic regurgitation, GCW—global constructive work, GWE—global work efficiency, GWI—global work index, GWW—global waste work, LVEF—left ventricular ejection fraction, PGA—pressure gradient across aortic valve.

## Data Availability

Data are available upon reasonable request to authors.
